# Intrinsic apoptotic effect of Anatolian honeybee (*Apis mellifera anatoliaca*) venom promoted with mesoporous silica nanocarriers

**DOI:** 10.55730/1300-0152.2736

**Published:** 2024-12-30

**Authors:** Batuhan ORMAN, Aylin KOÇ, Didem ŞEN KARAMAN, Ayşe NALBANTSOY

**Affiliations:** 1 Department of Biotechnology, Graduate School of Natural and Applied Sciences, Ege University, İzmir, Turkiye; 2 Department of Biomedical Engineering, Graduate School of Natural and Applied Sciences, İzmir Kâtip Çelebi University, İzmir, Turkiye; 3 Department of Biomedical Engineering, Faculty of Engineering and Architecture, İzmir Kâtip Çelebi University, İzmir, Turkiye; 4 Department of Bioengineering, Faculty of Engineering, Ege University, İzmir, Turkiye

**Keywords:** Bee venom, *Apis mellifera anatoliaca*, anticancer biomolecule, mesoporous silica nanoparticles

## Abstract

**Background/aim:**

The use of bee products or treatment with bees, as a complement to conventional medicine is attracting considerable attention in cancer research. Although discoveries related to the potential anticancer effects of bee venom are increasing, the unstable nature of venom biomolecules remains a limiting factor for their usage. In this study, we employed mesoporous silica nanocarriers (MSNs) to provide precise dosing and prevent carriers from biomolecule degradation thanks to the outstanding loading capacity provided by the pores, excellent chemical and biological robustness, and ability to improve bioavailability.

**Materials and methods:**

MSNs were synthesized and physicochemical characterizations were carried out. The cytotoxicity of *Apis mellifera anatoliaca* bee venom and venom-complexed MSNs (MSNs@Venom) were determined for the MDA-MB 231, PC3, and HeLa cancer cell lines and the cytotoxicity of pristine MSNs was investigated for the HEK-293 and CCD34-Lu cell lines. The cellular uptake of MSNs@Venom by PC3 and MDA-MB 231 cells was investigated by fluorescence microscopy and flow cytometry. The apoptotic effect on cancer cells was examined by flow cytometry.

**Results:**

The MSNs exhibited significant cellular uptake of MSN by the PC3 and MDA-MB 231 cell lines, resulting in a 1.5-fold enhancement in the apoptotic effect of venom on the PC3 cell line when combined with MSNs, compared to cells exposed alone to venom.

**Conclusion:**

MSNs could effectively be taken up by MDA-MB 231 and PC3 cancer cells, enhancing the action of bee venom by the particle-mediated delivery. MSNs@Venom have the potential to offer cost-effective complementary and innovative cancer treatment options.

## 1. Introduction

The use of bee products, and particularly bee venom, in apitherapy for complementary or adjunctive cancer treatments has attracted interest due to the natural origins of these products and their cost-effectiveness. This affordability and accessibility allow them to serve as advantageous alternatives or supplements to traditional treatments. The use of bee venom dates back over 6000 years to ancient Egypt, where it was used for various medicinal purposes. Similarly, the Greeks and Romans recognized the therapeutic properties of bee products (Hellner et al., 2008). Honeybee venom has historically served as a traditional medicine (Son et al., 2007). Reports indicate that bee venom and its primary component, melittin, exhibit greater cytotoxic effects against various cancer cells, including prostate (Badawi, 2021), glioblastoma (Sisakht et al., 2017), lung (Gao et al., 2018), and breast (Duffy et al., 2020) cancer cells, compared to normal cells. Previous studies have attributed its antitumor effects to the three main components of melittin, phospholipase A2, and apamin, although high doses may cause neurotoxicity, hemolysis, and inflammation (Oršolić, 2012). Honeybee venom and its constituents modulate various genes and proteins, including caspases, death receptors, NF-κB, VEGF, HIF-1α, p53, and metalloproteinases, implicated in regulating apoptosis, proliferation, metastasis, angiogenesis, motility, and invasion (Putz et al., 2006; Li et al., 2009; Rady et al., 2017). Although the intricate mechanisms of action against tumor cells remain largely unknown, these findings underscore the potential of bee venom and its components.

Despite increasing discoveries in the context of venom-based applications, the instability of biomolecules limits their bioavailability and raises concerns about nonspecific toxicity. Bee venom has demonstrated adverse effects in normal cells (Gülmez et al., 2017; Cherniack and Govorushko, 2018; El-Didamony et al., 2022; Małek et al., 2023). Further research is needed to ascertain whether the cytotoxic effects of bee venom against normal cells can be mitigated while enhancing its therapeutic efficacy. To overcome the limitations, combining bee venom with nanosized drug carriers has emerged as a promising strategy (Xu et al., 2019; Khalil et al., 2021). Nano drug carriers play crucial roles in various biomedical fields with antimicrobial, anticancer, antiviral, and biocidal applications, enhancing the solubility, stability, functionality, and delivery of bioactive drugs or compounds in vivo and in vitro (Rout et al., 2018). Among various nanocarriers, mesoporous silica nanoparticles (MSNs) stand out due to their unique physicochemical properties, such as exceptional chemical and biological robustness and cellular interaction capabilities, making them ideal for the loading, protection, and prevention of premature degradation of biomolecules (Şen Karaman and Kettiger, 2018; Selvarajan et al., 2020). Extensive documentation has shown that loading biomolecules into the pores of MSNs enhances their stability and activity, as the inorganic framework effectively prevents protein denaturation (Küçüktürkmen and Rosenholm, 2021). Increasing the pore size of MSN facilitates biomolecule loading into pore channels; additionally, the tunable surface chemistry of MSNs presents a significant advantage in utilizing them for biomolecule delivery (Cha et al., 2018). Noncovalent interactions such as hydrogen bonding, electrostatic interactions, and π-stacking are commonly utilized to facilitate the delivery of biomolecules using MSNs (Yang et al., 2012). Researchers have modified the surface chemistry of MSNs to assess the degree of biomolecule (e.g., proteins) encapsulation, and the efficacy of biomolecule loading is primarily influenced by the surface chemistry of MSNs and the nature of the proteins involved. Notably, the protein loading process can be achieved rapidly within 20 min, reaching maximum capacity (Tu et al., 2016).

Amine-modified mesoporous silica nanoparticles (MSNs@Venom) were selected in the present study for their ability to enhance cellular uptake and increase the therapeutic efficacy of bee venom. The amine groups on MSNs facilitate the electrostatic adsorption of bee venom, allowing for more targeted delivery to adenocarcinoma cells, which are characterized by high metastasis rates. In vitro investigations were conducted to assess their cytocompatibility and cellular uptake in various adenocarcinoma cell lines, including prostate cancer cells (PC3), breast cancer cells (MDA-MB 231), and cervical cancer cells (HeLa). The findings indicated that MSN-mediated delivery significantly enhanced the apoptotic effects of honeybee venom compared to its free form, with MSNs@Venom showing increased selectivity for the PC3 cell line compared to MDA-MB 231. To the best of our knowledge, this study is the first to evaluate the cytotoxic effects and apoptotic activity of amine-modified MSNs loaded with honeybee venom, both with and without MSN delivery, highlighting the in vitro selectivity of these MSNs across different adenocarcinoma cell lines

The significant increase in apoptosis observed in these cancer cell lines compared to free honeybee venom suggests that amine-modified MSNs have the potential to enhance therapeutic efficacy.

## 2. Materials and methods

### 2.1. Synthesis and characterization of mesoporous silica nanoparticles and honeybee venom complexation with mesoporous silica nanoparticles

The synthesis of MCM-41-type MSNs was performed using a sol-gel protocol detailed in our previous studies (Desai et al., 2014). Cetyltrimethylammonium bromide (CTAB; Sigma, St. Louis, MO, USA), used as the structure-directing agent (SDA), was initially dissolved in a basic ethanolic aqueous solution. Subsequently, tetraethyl orthosilicate (TEOS; Merck, Rahway, NJ, USA) was added as the silica source. The mixture was then stirred continuously overnight. The synthesis solution’s molar composition was set to 1 TEOS:0.122 CTAB:0.31 NaOH:72.3 EtOH:946 H_2_O and the resulting samples were labeled as MSNs. Fluorescein isothiocyanate (FITC; Sigma, USA), an amine-reactive fluorophore, was used for the fluorescent labeling. A prereaction mixture of FITC and aminopropyl triethoxysilane (APTES; Sigma) in ethanol (2 mL) at a 1:3 molar ratio was stirred for 2 h to incorporate the FITC into the silica matrix. The prereacted mixture was added to the synthesis solution just before adding TEOS, keeping an APTES-to-TEOS molar ratio of 10:100. Synthesis occurred overnight at a controlled temperature of 33 °C. After the reaction, the SDA was removed by sonication extraction performed three times in an ethanolic NH_4_NO_3_ solution, yielding FITC-labeled particles, referred to as F-MSNs.

Bee venom solutions, provided by Ar-Sum Apimak (Ankara, Türkiye), were dissolved in phosphate buffer (pH 7.2, 12 mM) for complexation with the MSNs in proportions ranging from 1% to 100% by weight. The solution of nanoparticles and bee venom was stirred at 400 rpm for 1 h at room temperature to allow for adsorption. Subsequently, the solution was centrifuged at 8000 rpm for 10 min and the loading capacity of the bee venom peptides in the supernatant was measured using a BCA kit according to the manufacturer’s instructions (Thermo Fisher Scientific, Waltham, MA, USA). Bovine serum albumin (BSA) was used as the protein standard. The bee venom-loaded samples are henceforth referred to as MSNs@Venom.

The characterization of the nanoparticles was accomplished by determining their size and distribution using dynamic light scattering (DLS), assessing their zeta potential to infer surface charge properties, and examining their surface morphologies with scanning electron microscopy (SEM). Initially, the nanoparticles were dispersed in HEPES buffer solution (pH 7.2, 25 mM; Sigma) at a concentration of 200 μg/mL and subjected to ultrasonic treatment in a bath for 20 min to achieve a homogeneous distribution. Subsequently, the size distribution and zeta potential of the nanoparticles were measured using a Zetasizer device (Malvern PANalytical, Malvern, UK). The DLS technique, facilitated by Zetasizer device, allowed for the precise determination of the nanoparticles’ sizes and distributions, and the same device was also employed to measure their zeta potential, providing insights into their surface charge characteristics.

The morphology of the obtained nanoparticles was determined by SEM imaging. Sample suspensions, dispersed in approximately 100 μL of ethanol, were dripped onto microscope slides and left to dry in a dust-free environment, and all samples were screened.

### 2.2. In vitro cell culture investigations

In vitro testing of the bee venom, MSNs@Venom, and pristine MSNs was conducted on the nontumorigenic human cell lines CCD34-Lu and HEK-293, sourced from fibroblast and epithelial tissues, respectively. The therapeutic effects of the designed nanoparticles (MSNs@Venom) and the constituents (pristine MSNs and bee venom) were further investigated with tumorigenic cell lines possessing varying tumorigenicity profiles to assess their potential in targeted cancer therapy. Specifically, the PC3 cell line, known for its high tumorigenic potential, was chosen to evaluate aggressive cancer behaviors, highlighting the effectiveness of MSNs@Venom in more malignant scenarios (Zhang and Waxman, 2010). Conversely, the HeLa cell line, characterized by its poor tumorigenic capabilities, provided a contrast on the spectrum of cancer cell aggressiveness, allowing for a comprehensive analysis of therapeutic outcomes across different cancer types (Oh et al., 2022). These investigations were also performed with adenocarcinoma cell lines MDA-MB 231, PC3, and HeLa to encompass a broad range of cellular responses to the treatment modalities. In the in vitro investigations, cells that had not been subjected to any treatment served as the control group.

Cells were obtained in stock from the ATCC (Manassas, VA, USA). The cells were removed from the stock and incubated in a mixture of Dulbecco’s modified Eagle medium/Nutrient Mixture F-12, 10% fetal bovine serum, 1% L-glutamine, 0.1% penicillin-streptomycin, and 1 mM HEPES (GIBCO, Thermo Fisher Scientific, USA) in a CO_2_ incubator at 37 °C to maintain their continuity. Cells were checked for mycoplasma by 4′,6-diamidino-2-phenylindole dihydrochloride (DAPI) staining (Sigma Aldrich, St. Louis, MO, USA). The medium of the cells was changed every 2 days and the cells were passaged when they covered the surface of the flask. For the passaging process, the surface of the flask was thoroughly washed with 2 mL of phosphate-buffered saline (PBS; GIBCO, USA) after the culture medium was aspirated. Subsequently, 1 mL of trypsin/EDTA (0.25%; GIBCO) was added and the cells were removed from the flask by incubation for 5 min at 37 °C in a CO_2_ incubator. Cells were then collected with 5 mL of medium. Culturing was continued with 10–15 mL of fresh medium for a 75-cm^2^ flask.

### 2.3. In vitro cytocompatibility of pristine MSNs with human cell lines and potential anticancer effects of MSNs@Venom against human adenocarcinoma cell lines

The noncytotoxic effects of the MSN carriers were investigated using healthy human CCD34-LU and HEK-293 cells to affirm the in vitro cytocompatibility of the nanocarriers. Furthermore, the cell viability of PC3, HeLa, and MDA-MB adenocarcinoma cells treated with MSNs@Venom was investigated to evaluate the potential anticancer effects of the designed venom–nanoparticle complex. For both investigations, the 3-(4,5-dimethylthiazol-2-yl)-2,5-diphenyltetrazolium bromide (MTT; Sigma) assay was used. Cells were seeded in 96-well microplates at 1 × 10^4^ cells/well and incubated for 24 h at 37 °C in an atmosphere of 5% CO_2_ and 95% humidity. For both investigations, MSNs and MSNs@Venom dispersions (5–100 μg/mL) in cell culture media were introduced and incubated for 24 and 48 h. Comparative treatments with pristine bee venom were also performed for the adenocarcinoma cell lines as a solution with equivalent dosing to that of the MSNs@Venom. The treated cells were examined with a microscope, the medium was aspirated, and cell media were removed by washing their surfaces with PBS. Subsequently, 100 μL of medium and 10 μL (5 mg/mL) of MTT were added to each well and the wells were incubated for 2–4 h at 37 °C, protected from light. The medium was then removed, 150 μL of DMSO was added to dissolve the formazan crystals, and absorbance was read with a UV-VIS microplate reader (The BioTek Synergy HTX, Vermont, U.S.) at a wavelength of 570 nm. The % viability values were determined by comparison with the controls. GraphPad Prism 5 (GraphPad Inc., San Diego, CA, USA) was used for IC_50_ calculations.

### 2.4. In vitro intracellular uptake of MSNs by adenocarcinoma cancer cell lines

Intracellular uptake of MSNs and MSNs@Venom by the PC3 and MDA-MB 231 cell lines was investigated through flow cytometry analysis and fluorescent microscopy imaging. Preliminary results demonstrated a substantial decrease in cell viability below 70% when these cells were treated with MSNs@Venom compared to the HeLa cell line, which suggests a more potent anticancer effect against PC3 and MDA-MB 231 cells. Consequently, these specific breast and prostate cancer cell lines were chosen for further cellular uptake studies. For in vitro maintenance, PC3 and MDA-MB 231 cells were cultured at volumes of 2 mL or 2.5 × 10^5^ cells/mL in 6-well plates for 24 h. The MSNs and MSNs@Venom were then incubated with the cells at concentrations of 10, 20, and 50 μg/mL for 4 and 24 h. Following incubation, the cells were thoroughly washed with PBS and trypsinized, and then they were centrifuged at 2000 rpm for 5 min. Cells were treated with 20 μg/mL trypan blue (Sigma) for approximately 5 min to quench the extracellular fluorescence from noninternalized MSNs. The experimental groups were resuspended in 500 μL of PBS for flow cytometry analysis with an Accuri C5 device (BD, Franklin Lakes, NJ, USA). Changes in green fluorescence were examined to trace the fluorescent dye-labeled MSNs. The highest cellular uptake condition for fluorescent microscopy imaging sessions, 50 μg/mL, was chosen for MSNs and MSNs@Venom. Cell seeding and incubation were performed on coverslips. After the incubation, cells were washed with 1 mL of PBS, and fixation was performed at −20 °C for 30 min with 1 mL of MeOH. Following methanol removal, cells were treated with 1 mL of 0.2% Triton X (VWR, Radnor, PA, USA) with shaking for 5 min to enhance the dye penetration. After subsequent PBS washes and dehydration with 70% and 100% ethanol, cells were stained with 10 μL (0.1 μg/mL) of DAPI and incubated at 4 °C for 30–35 min. Imaging was then performed using a DM4000 B LED fluorescent microscope (Leica, Wetzlar, Germany).

### 2.5. Determination of apoptotic effects of MSNs, bee venom, and MSNs@Venom

A staining method involving apoptotic cell marker FITC and conjugated annexin V (Invitrogen, Carlsbad, CA, USA) was used to examine PC3 and MDA-MB 231 cells exposed to pure bee venom, MSNs, and MSNs@Venom. PC3 and MDA-MB 231 cells in the logarithmic phase were seeded in 6-well plates at 2.5 × 10^5^ cells/mL and incubated for 24 h in a humid atmosphere with 5% CO_2_ at 37 °C. After the medium was aspirated, treatments of 50 μg/mL and 100 μg/mL MSNs, MSNs@Venom suspensions, and bee venom solution equivalent to the bee venom contents of the MSNs@Venom treatments of 50 μg/mL and 100 μg/mL were applied to the cells. Cells were incubated for 24 h (5% CO_2_, 37 °C, 95% humidity) with the specified treatment dosages. Apoptotic cells were then analyzed quantitatively by flow cytometry using an Invitrogen (USA) kit according to the manufacturer’s instructions. After washing the cells with PBS, they were removed by trypsinization. They were then washed 3 times with 500 μL of PBS and the parameters of 2000 rpm, 5 min, and 4 °C were applied in the precipitation processes. Following these procedures, 100 μL of binding buffer (1X) was added and 1 μL of FITC-conjugated annexin V was added to the suspended cells, which were incubated for 15 min in the dark. Cells were then washed two times with PBS, 200 μL of PBS was added, and measurements were taken by flow cytometry. IC_50_ values were calculated with GraphPad Prism based on nonlinear regression and dose-dependent inhibition analyses with the concentration of the administered doses against the response values calculated as % cell viability.

### 2.6. Statistical analysis

Data were obtained from three replicates of experiments. Statistical analyses were conducted with Graph Pad Prism 5 and Microsoft Office Excel. The significance of differences compared to controls was determined with one-way and two-way analysis of variance (ANOVA) and Dunnet’s multiple comparison test. The significance of the data was compared with Student’s t-test. In all tests, differences were considered significant at p < 0.05, p < 0.01, and p < 0.001.

## 3. Results

### 3.1. Physiochemical characterization of mesoporous silica nanoparticles

**Figure 1:**
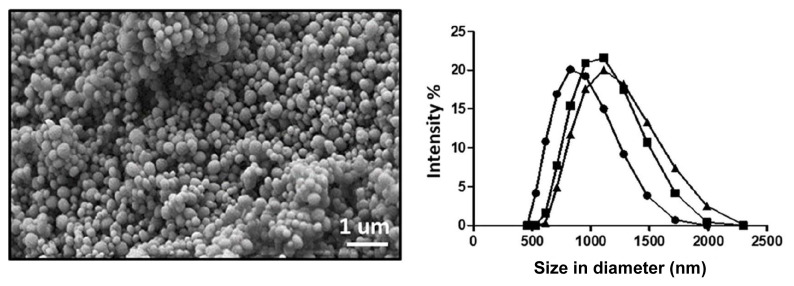
**a)** Scanning electron microscopy images of MSNs and **b)** hydrodynamic size distribution plot

Morphological study of the MSNs revealed spherically monodispersed particles by SEM as seen in Figure 1a. Furthermore, hydrodynamic size and zeta potential measurement values were obtained as 1115 ± 206.3 nm and +15 ± 4.43 mV, respectively, in HEPES buffer solution as presented in Figure 1b. The mismatch of the MSN sizes obtained from SEM images and DLS analysis can be ascribed to the low ζ-potential value of the MSNs, which is in the range of slightly low net values, indicating flocculation of the MSNs. In these investigations, samplings were performed at physiological pH of 7.2 (HEPES buffer solution, 25 mM) to mimic the preparation steps in nanoparticle suspension stock solution for in vitro studies.

### 3.2. Bee venom loading on mesoporous silica nanoparticles

**Figure 2:**
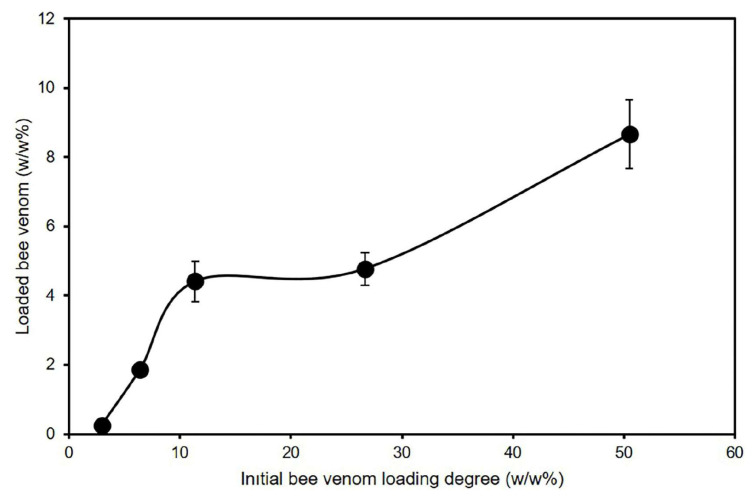
Adsorption isotherm of total venom protein on MSNs.

To investigate the complexation potential of the honeybee venom to be used in this study, adsorption of venom was ensured with amounts of venom corresponding to 1%–100% of the weight of MSNs. Since the effect of the venom is thought to be caused by the peptides it contains, the protein amounts in the supernatant were measured with BSA calibration using the Pierce BCA Protein Assay Kit (Thermo Fisher Scientific) and loading efficiency was calculated as presented in Figure 2. The plots showed that loading starting with 25 w/w% bee venom yielded a loading degree of approximately 5 w/w%, which was assigned as the highest loading efficiency. These preparations were chosen as optimal and were used for further in vitro investigations.

### 3.3. Determination of cytotoxic effects of bee venom, mesoporous silica nanoparticles, and venom-loaded silica nanoparticles

**Figure 3:**
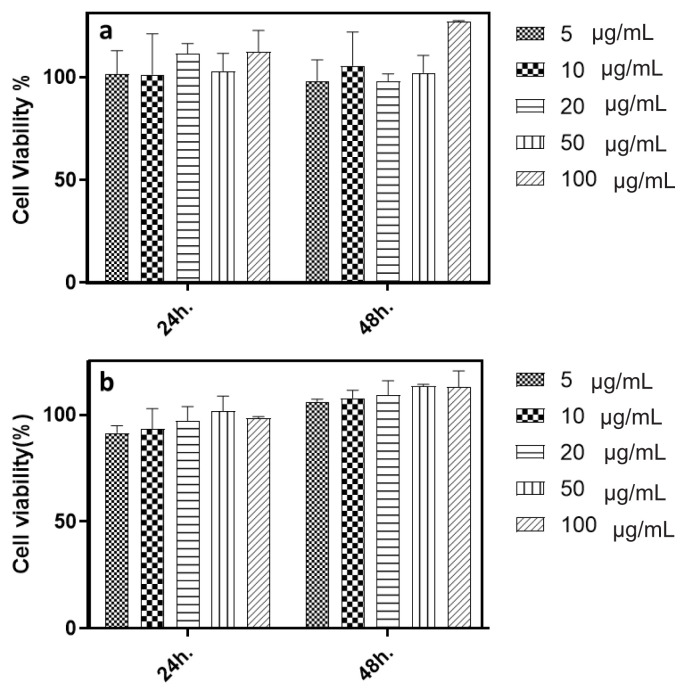
Effects of MSNs on cell line viability of **(a)** HEK-293 (human embryonic kidney cells) and **(b)** CCD34-Lu (healthy lung fibroblast cells).

The cytotoxic effects of the MSNs on the nontumorigenic HEK-293 and CCD34-Lu cell lines were determined by MTT assay and % viability graphs are presented in Figure 3. The cell lines were exposed to MSNs with increasing concentrations for 24 and 48 h. Considering both the times and the doses, no cytotoxic effects were observed.

**Figure 4:**
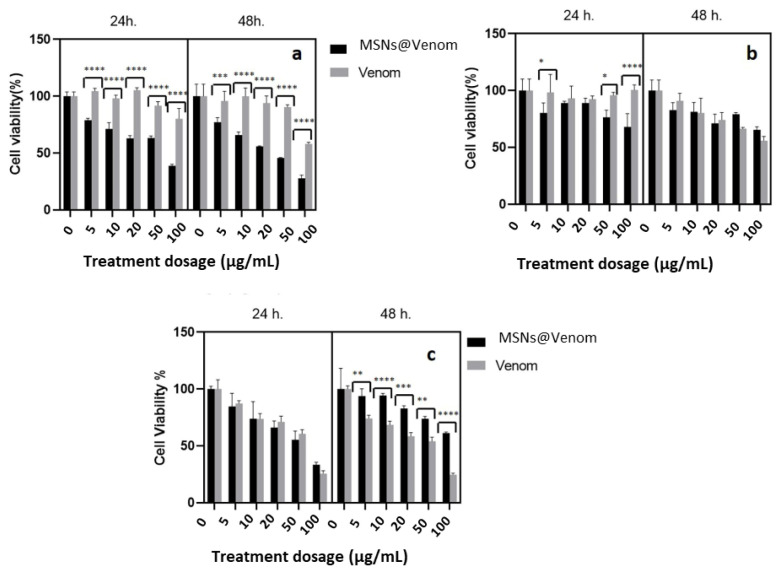
Cytotoxicity investigations of cells treated with MSNs@Venom and pristine venom in vitro for 24 and 48 h: **(a)** MDA-MB 231 breast cancer cells, **(b)** HeLa cervical cancer cells, and **(c)** PC3 prostate cancer cells. *: p < 0.02, **: p < 0.01, ***: p < 0.001, ****: p < 0.0001.

After establishing the safe dosage range for MSN nanocarriers, we performed in vitro cytotoxicity investigations with MSNs@Venom, MSNs, and bee venom solution at equivalent concentrations to the venom amount adsorbed by MSNs@Venom, comparing different concentrations among the tumorigenic MDA-MB 231, HeLa, and PC3 cancer cell lines. As presented in Figure 4, the cytotoxicity of MSNs@Venom and equivalent venom dosing depended on the concentration, incubation time, and cell line. MSNs@Venom showed the most dominant effect against MDA-MB 231 cells (Figure 4a). However, we observed no cytotoxic effect against HeLa cells. In PC3 cells, MSNs@Venom exerted an adverse effect on cell viability compared to treatment with venom alone.

IC_50_ values were calculated with GraphPad Prism by nonlinear regression and dose-dependent inhibition analyses were conducted for the concentration of administered doses against response values, yielding % cell viability results. As given in the Table, the amounts of adsorbed venom on MSNs were calculated with % loading efficiencies after calculating the IC_50_ values of the MSNs@Venom treatments. The estimation revealed that all cell lines were more susceptible to MSNs@Venom considering 24-h treatments. The required bee venom dosing could be reduced by almost 10-fold with the aid of MSN carriers for all cell lines in 24-h treatments. However, this finding was less clear for the PC3 and HeLa cell lines, especially when the incubation period was extended.

### 3.4. Quantitative measurement of *in vitro* cellular uptake of MSNs and MSNs@Venom by flow cytometry

**Figure 5:**
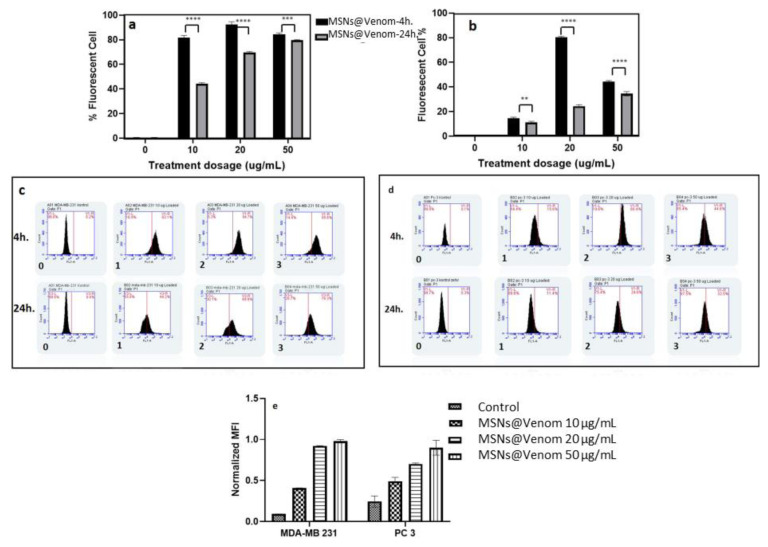
Cellular uptake comparison graphs for MSNs@Venom after 4 h and 24 h of treatment with increasing doses ranging between 10 and 50 μg/mL for **(a)** MDA-MB 231 and **(b)** PC3 cells (*: p < 0.05, **: p < 0.028, ***: p < 0.0006, ****: p < 0.0001). Histogram profiles of **(c)** MDA-MB 231 and **(d)** PC3 cell lines incubated with MSNs@Venom for 4 h and 24 h. **(e)** Normalized mean fluorescence intensity at 4 h and 24 h for MSNs@Venom (0–50 μg/mL) in MDA-MB 231 and PC3 cells.

In cellular uptake investigations, the PC3 and MDA-MB 231 cell lines were selected due to their reduced cell viability following MSNs@Venom treatment, which indicates enhanced activity, as shown in Figure 4. Flow cytometry analysis revealed higher cellular uptake of MSNs@Venom for shorter incubation periods compared to longer incubation periods for both MDA-MB 231 and PC3 cell lines, as illustrated in Figures 5a–5e. Extending the time duration yielded a decrease in florescent cell population percentage in PC3 cells, as seen in Figure 5b. When we evaluated the results while considering the normalized mean fluorescent intensity values for the PC3 and MDA-MB 231 cell lines with increasing concentrations, similar trends were observed for PC3 and MDA-MB 231 cells, as presented in Figure 5e. These findings are also supported by the histogram profiles presented in Figures 5c and 5d.

**Figure 6:**
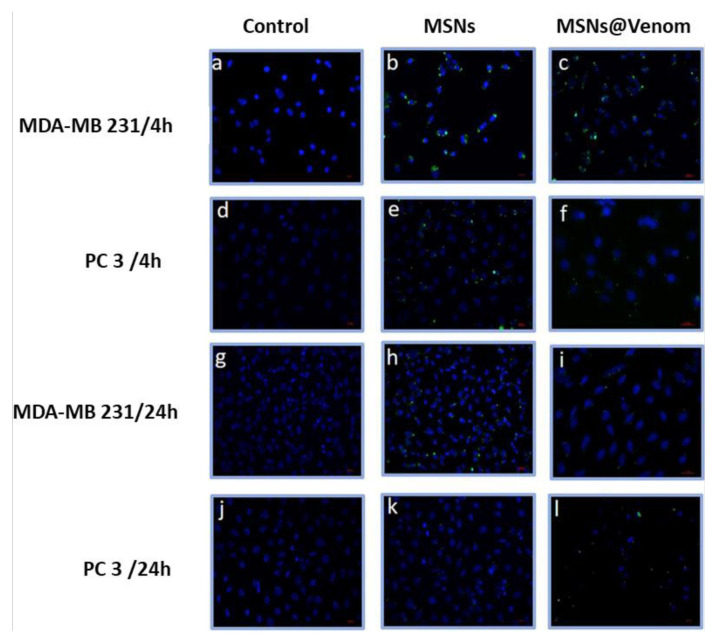
Fluorescent microscopy images showing the intracellular uptake of FITC-labeled MSNs (50 μg/mL) and MSNs@Venom (50 μg/mL) (green) in MDA-MB 231 and PC3 cell lines incubated for 4 and 24 h. The MSNs and MSNs@Venom were localized closely around the nucleus in MDA-MB 231 and PC3 cells. Nuclei were stained with DAPI (blue). Scale bar: 20 μm.

In Supplementary Figures S1a–S1d, we illustrate the cellular uptake of MSNs without venom loading, which was evaluated to ensure whether venom loading on MSNs could cause inhibition in cellular uptake. Supplementary Figure S clearly shows that venom adsorption on MSNs had a predominant effect on cellular internalization at lower concentrations, but when the concentration of the MSNs increased to 100 μg/mL, the normalized mean fluorescence intensity (MFI) values showed the induction of cellular uptake. This effect was more dominant in the MDA-MB 213 cell line compared to PC3 cells. Subsequently, the intracellular uptake of MSNs and MSNs@Venom was investigated using fluorescence microscopy images for a dosage of 50 μg/mL. As seen in Figure 6, green fluorescently labeled nanoparticles around the nuclei of cells were stained with DAPI in 4-h and 24-h applications. Extending the time duration caused a decrease in the visualization of MSNs@Venom in the MDA-MB 231 cell line, whereas for PC3 cells, MSNs@Venom could still be observed around the cell nuclei as green particles.

### 3.5. In vitro apoptotic activity investigation of MSNs@Venom

**Figure 7:**
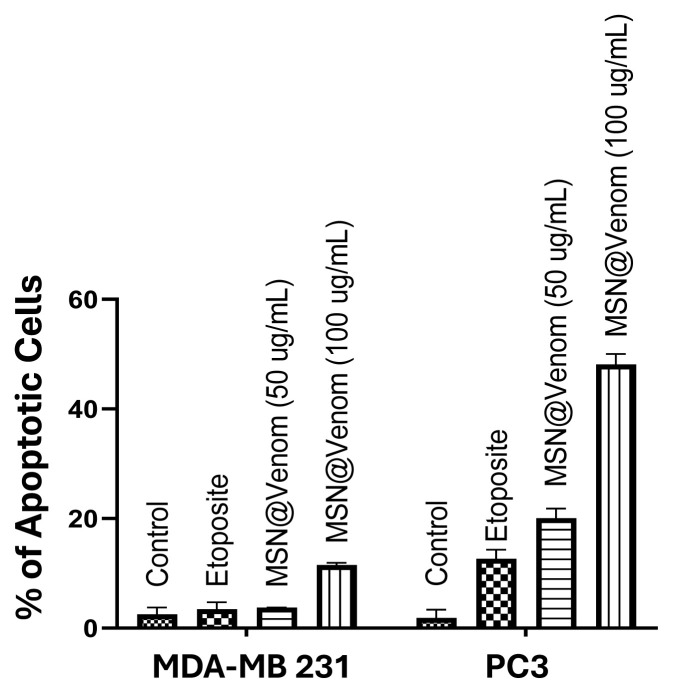
Percentage of apoptotic cells indicated as a proportion of the cell population for each treatment in MDA-MB 231 and PC3 cells.

Apoptotic activity investigations were performed for 50 and 100 μg/mL MSNs@Venom, pristine MSNs, and bee venom solution at a concentration equivalent to the venom amount adsorbed for 50 μg/mL and 100 μg/mL treatments of MDA-MB 231 and PC3 cells. The percentage of apoptotic cells was comparatively evaluated. Figure 7 shows that the strongest apoptotic activity was achieved in the PC3 cell line, especially with the increasing dosage regime of 50 and 100 μg/mL. However, the apoptotic cell percentage remained low for the MDA-MB 231 cell line.

## 4. Discussion

Due to the known anticancer effects of bee venom arising from its active components, this study aimed to increase the effectiveness of the venom of Anatolian honeybee, which is widely distributed in Türkiye, by adsorbing it onto MSN drug carriers. The literature confirms that MSNs are advantageous for providing increased permeability and retention at tumor sites together with the possibility of increased passive targeting with positively charged MSNs (Tarn et al., 2013). In addition, MSNs with highly positive net surface charges could provide the “proton sponge effect,” increasing the acidity in lysosomes as a result of the release of protons by cationic lipid, peptide, or synthetic materials on the surface, with ion flow to endosomes and excessive water entry. The increased osmotic pressure and the rupture of membranes releases the carried therapeutics into the cytoplasm (Baeza et al., 2016; Wojnilowicz et al., 2019). Accordingly, in this study we worked with MSNs possessing net positive charge values of 15 ± 4.43 mV according to zeta potential analysis. In our laboratory investigations, however, we also observed that the zeta potential value showed slight net surface charge increments after bee venom adsorption on MSNs. From previous observations, we know that the most dominant protein corona formation could be obtained for positively charged particle surfaces, which could be viewed as a drawback for both cellular uptake and the endosomal escape pathway (Şen Karaman et al., 2014; Huang et al., 2016). However, the adsorption of bee venom on MSNs may lead to the prevention of protein corona formation. In a previous study, researchers modified the protein corona of synthetic polymer nanoparticles, revealing that the optimized nanoparticles exhibited selectivity for venomous PLA2 over more prevalent serum proteins, lacked cytotoxicity, and displayed significantly prolonged dissociation rates from PLA2 (O’Brien et al., 2016). These findings indicate its potential efficacy as an in vivo venom sequestrant and as a generalized lipid-mediated toxin sequestrant. Furthermore, melittin, as a main component of bee venom, can regulate the cell cycle, change the permeability of cell membranes, inhibit proliferation and migration, and promote endogenous/exogenous apoptosis and autophagy and other regulatory cell death modes to cause cell death (Mirzaei et al., 2021).

The effects of MSNs on nontumorigenic HEK-293 and healthy CCD34-Lu cells were examined by MTT assay. No toxic effects were observed in MSNs with FITC-APTES after 24 or 48 h of treatment. It is generally accepted that silica nanoparticles are nontoxic, but it has been reported that toxic effects may occur depending on the specific properties of nanoparticle size and the reactive surface groups (Selvarajan et al., 2020). In cytotoxicity investigations of the MSNs, our findings were in parallel with the literature, as researchers have previously investigated and compared the size-dependent cytotoxicity of MSNs against different cell lines. MSNs of 100–150 nm in size showed the highest rates of tumor targeting and uptake compared to smaller (<100 nm) or larger (>300 nm) sizes (Kim et al., 2014). As seen in the present study, the hydrodynamic size of the MSNs is greater than 500 nm and they contributed to limited cytotoxicity, which might be due to low cellular uptake. Therefore, it is critical to observe the cellular uptake of such particles with *in vitro* investigations, which we performed for both pristine MSNs and MSNs@Venom. Cell types are also critically important in influencing such results, and we performed our investigations utilizing one of the commonly used cell lines, HEK-293, and an uncommon cell line, CCD34-Lu, constituting kidney and lung cells, respectively. The differential sensitivity of specific cell types, p53 competency, and various cellular uptake mechanisms may be listed as the dominant factors leading to differences in cytotoxic effects (Ahmadi et al., 2022). In the literature, spherical MSNs with sizes of 190, 420, and 1220 nm were reported to have cytotoxicity dependent on size, with smaller MSNs (190 nm) being more cytotoxic than larger MSNs (1220 nm) due to decreased endocytosis with larger sizes (He et al., 2009). These findings are in parallel with the results of our investigations, as we did not observe any cytotoxic effects of the MSNs (with hydrodynamic size of 1115 ± 206.3 nm) against the tested cells. Our observations revealed that MSN nanocarriers at concentrations of 5–100 μg/mL did not cause any cytotoxic effects in nontumorigenic HEK-293 (human embryonic kidney cells) and CCD34-Lu (healthy lung fibroblast cells) cell lines. Subsequently, bee venom was adsorbed on these MSNs and the loading efficiency of the venom on MSNs was calculated. Our findings revealed that 5 w/w% bee venom in relation to the weight of the MSNs could be adsorbed on MSN carriers with the highest results, as presented in Figure 2. Although it has been shown that the stability and effects of bee venom and melittin are increased with various nanoparticle systems, there are limited studies in the literature on MSNs in this regard. MSNs are slowly emerging as promising carriers for the targeted delivery of venom peptides in cancer therapy, addressing challenges of instability, toxicity, and poor bioavailability (Gauri V. Deodhar et al., 2017; Mirzaei et al., 2021; Chary et al., 2024). MSNs have also been successfully used to deliver venom proteins while preserving their activity (Baudou et al., 2020) and to immobilize enzymes with improved thermal stability and activity (El-Boubbou K et al., 2012; Xu et al., 2019). Misra et al. (2015) developed a methodology from in silico to in vitro to synthesize a precisely defined, self-assembled, rigid-cored polymeric (“polybee”) nanoarchitecture for the controlled delivery of melittin. Through a series of sequential experiments, they examined how nanoscale chemistry affects the delivery of venom toxins for cancer remission and aids in addressing problems of systemic integrity and cellular toxicity. Their findings suggested that melittin may have anticancer properties through its potential to be involved in DNA association and dissociation processes. Furthermore, Ravindra et al. (2004) showed that MSNs can enhance protein stability and delivery. In their study, protein encapsulation in MSNs significantly increased thermal stability, with an increase of 30 °C in the unfolding temperature. Controllable morphologies and functionalizable surface chemistries make MSNs well suited for cancer treatment applications (Barkat et al., 2021). Our investigations with bee venom-loaded MSNs (MSNs@Venom) did not induce any adverse effects on the colloidal properties of the MSNs and the net surface charge value of the MSNs@Venom was increased to 21 mV at a physiological pH of 7.2 with improved colloidal dispersibility of MSNs down to hydrodynamic sizes of 500 nm. Studies in the literature show that snake venom and various peptide biomolecules can also be efficiently loaded and encapsulated into MSNs (He et al., 2009; Şen Karaman and Kettiger, 2018; Mirzaei et al., 2021; Ahmadi et al., 2022). In our study, loading degrees of 4.77 w/w% were obtained for relatively low yields compared to the literature. This could be attributed to the employed method for physical adsorption, which was achieved in phosphate buffer solution. It may be due to the repulsive forces originating from the cationic nature of melittin (Rady et al., 2017), which constitutes the major component of venom, although it is relatively close to neutral. A previous study involving an antimicrobial cationic peptide supports this finding, as that study indicated that positively charged nanoparticles exhibited greater interactions with the membrane despite a lower yield (Braun et al., 2016). This is in line with the low loading amount due to the structure of melittin and the loading of other proteins and peptides of venom onto the MSNs. As part of the in vitro analysis, cellular internalization investigations and the intracellular activities of venom-loaded MSN revealed that MSNs@Venom cellular uptake takes place during incubation periods of 24 and 48 h. The normalized MFI values presented in Figure 5e confirmed that extending the incubation time contributed to cellular uptake with increased concentrations of MSNs@Venom with similar trends observed for increased concentrations of MSNs for both MDA-MB 231 and PC3 cells. Additionally, exposure of the selected tumorigenic cell lines to MSNs@Venom led to reduced bee venom dosing for the IC_50_ value compared to treatments with pristine venom. Our findings align with previous research on chitosan nanoparticles conjugated with bee venom to enhance their anticancer activity and reduce their toxicity in nontarget cells (Moselhy Walaa et al., 2014). It was concluded in that study that the nanoparticles enhanced the desired toxic effect of bee venom, arresting the cell cycle in HePG2 and PC3 cells. Treatment of MDA-MB 231 cells in the present study yielded a nearly 10-fold decrease in the required dose for inhibiting cell viability in both 24-h and 48-h treatments compared to treatment with pristine bee venom. However, for the PC3 cells, this effect was limited to 24 h, which could be due to metabolic features of the PC3 androgen-independent human prostate cancer cell line (Zacharias et al., 2017). It could also be related to the rapid release of the bee venom components from MSN carriers, influencing the components of bee venom that cannot act outside the cells due to its complex structure. Further investigations of the apoptotic activity of MSNs@Venom against two different cancer cell lines showed the predominance of specific cell death in PC3 cells. However, in the MDA-MB 231 cell line, levels of apoptotic activity were significantly lower compared to the PC3 cell line. The strong apoptotic effects of MSNs@Venom, particularly against the PC3 cell line, can be attributed to the synergistic interactions between the bioactive components of bee venom and the enhanced delivery efficiency provided by MSNs. Bee venom is rich in melittin, exhibits potent cytolytic activity, and triggers apoptosis through multiple mechanisms including the generation of reactive oxygen species, disruption of mitochondrial membrane potential, and the release of cytochrome c, leading to the activation of apoptotic cascades (Ip et al., 2008). In PC3 cells, melittin’s robust interaction with cancer cell membranes leads to heightened sensitivity and apoptotic responses, with a substantial decrease in the viability of PC3 cells at a minimal melittin concentration of 0.01 μg/mL indicating the peptide’s potent cytolytic activity and suggesting its potential as a compound for antitumor therapy (Khalikov et al., 2022). In previous studies of the anticancer effects of bee venom against MDA-MB 231 cells, bee venom induced apoptosis through mechanisms such as DNA fragmentation and protein denaturation (Jung et al., 2018; Borojeni et al., 2020; Sevin et al., 2023). The therapeutic effects of bee venom could be maximized while minimizing off-target apoptotic effects (Badawi, 2021). Differences between studies might be due to specific dominant effects of venom on particular cell membranes or the inhibited membrane effect of bee venom due to cellular uptake of MSNs@Venom in this study, which might have caused an inability to reach sufficient concentrations to trigger intracellular pathways.

The present study indicates that the utilization of positively charged MSNs not only promotes increased cellular uptake but also aids in the inhibition of protein corona formation, which reflects the biological significance of our work. This ability of the nanoparticles may enhance biodistribution and retention at the tumor site, which are essential for achieving enhanced permeability and retention effects in vivo. Moreover, melittin, as the bioactive component of bee venom, is known to regulate the cell cycle and trigger apoptosis, autophagy, and other mechanisms of regulated cell death, making MSNs@Venom multifaceted instruments for anticancer applications. Together with the biological significance of our findings, we anticipate that our results will offer clinical implications that highlight the potential of MSNs as efficient carriers for enhancing the therapeutic value of bee venom. The reduced dosing requirements for bee venom obtained in this study, and particularly for MDA-MB 231 cells, emphasize its potential in minimizing systemic toxicity as a critical factor for clinical translation. The ability of MSNs@Venom to reduce the IC_50_ value by nearly 10-fold offers promising avenues for targeted cancer therapy, enabling more effective dose management. Additionally, the selective apoptotic effect observed in the PC3 prostate cancer cell line indicates the possibility of tailoring this approach to specific cancer types based on cellular vulnerabilities and metabolic profiles. These findings align with the broader strategy of enhancing tumor selectivity while preserving healthy tissue, a cornerstone of modern oncology.

## 5. Conclusion

In this study, bee venom collected from *Apis mellifera anatoliaca* was adsorbed onto mesoporous silica nanocarriers (MSNs@Venom) and characterization was performed. The mesoporous silica based nanocarrierswas utilized to improve the previously confirmed apoptotic activity of bee venom through particle-mediated delivery. When tumorigenic cancer cells were treated with MSNs@Venom, cell growth inhibition was enhanced compared to the effects of pristine bee venom, and this effect could further enhance the apoptotic activity. The bee venom retained its activity in the tested tumorigenic cancer cell lines upon particle loading and cellular uptake. Taken together, our results show that the MSNs can be effectively internalized by MDA-MB 231 and PC3 cancer cells, further enhancing the action of the bee venom by particle-mediated delivery utilizing MSNs as a carrier system. These findings provide valuable insights into the differential sensitivity of cancer cell lines to bee venom and its components, underscoring the importance of delivery strategies such as MSNs@Venom in enhancing therapeutic efficacy while addressing potential limitations and paving the way for future advancements in targeted cancer therapies. In future studies, explanation of the molecular mechanisms of action of bee venom against cancer, clarification of the contents of the venom, and examination of the mechanisms of action of its components will enable the development of new apitherapy strategies utilizing MSN nanocarriers.

## Supplementary Document

Intrinsic Apoptotic Effect of Anatolian Honeybee (Apis mellifera anatoliaca) Venom Promoted with Mesoporous Silica Nanocarriers

Figure S MSN cellular uptake comparison graphs after 4 hours and 24 hours treatment with ascending dosing range between 10–50 ug/mL a) MDA-MB 231 b) PC3 (*p<0.05, **p<0.028, ***p<0.0006 ****p<0.0001). Histogram profiles of c) MDA-MB-231 and d) PC3 cell lines incubated with MSN for 4 hours and 24 hours. e) Normalized mean fluorescence intensity (MFI) of 24 h. MSN (0–100 μg mL−1) incubated MDA-MB 231 and PC3 cell lines

## Figures and Tables

**Table:** IC50 (μg/mL) estimations for bee venom dosing via MSNs@Venom and pristine bee venom for the selected cell lines.

	MDA-MB 231	PC3	HeLa
**Bee Venom – 24h**	>4.41	1.95	ND
**Bee Venom – 48h**	>4.41	1.40	>4.41
**MSN@Venom-24h.**	0.44	0.07	0.47
**MSN@Venom-48h.**	0.54	ND	ND
