# Supplementary figures and images for: Intrinsic apoptotic effect of Anatolian honeybee (Apis mellifera anatoliaca) venom promoted with mesoporous silica nanocarriers
Source: Turk J Biol. 2024 Dec 30;49(2):185–97. doi: 10.55730/1300-0152.2736 (PMC12068665; doi:10.55730/1300-0152.2736)

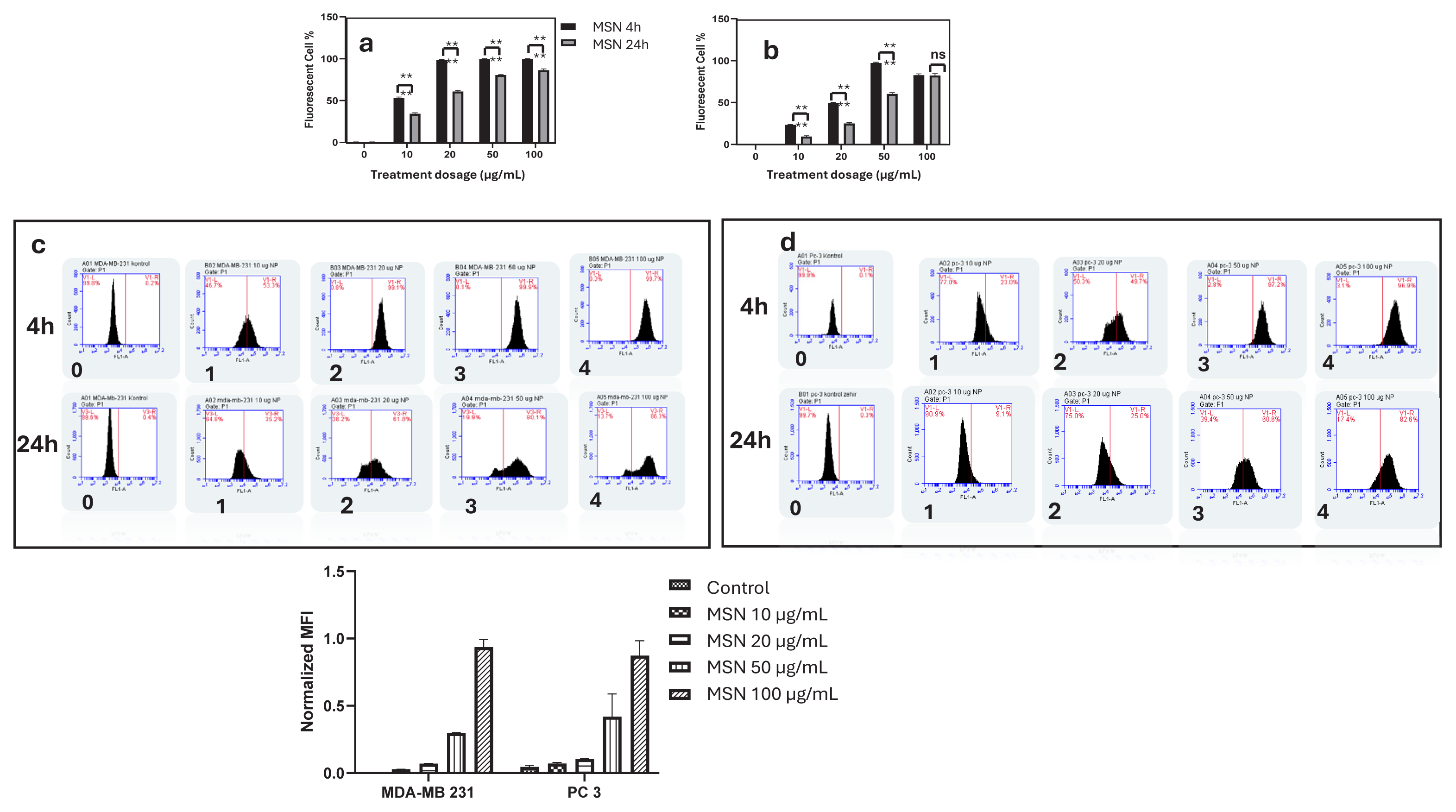

Supplement: Figure S — MSN cellular uptake comparison graphs after 4 hours and 24 hours treatment with ascending dosing range between 10–50 ug/mL a) MDA-MB 231 b) PC3 (*p<0.05, **p<0.028, ***p<0.0006 ****p<0.0001). Histogram profiles of c) MDA-MB-231 and d) PC3 cell lines incubated with MSN for 4 hours and 24 hours. e) Normalized mean fluorescence intensity (MFI) of 24 h. MSN (0–100 μg mL−1) incubated MDA-MB 231 and PC3 cell lines [file tjb-49-02-185s1.tif]
